# Exploring the Perceived Impact of the COVID-19 Pandemic Social Distancing Measures on Athlete Wellbeing: A Qualitative Study Utilising Photo-Elicitation

**DOI:** 10.3389/fpsyg.2021.624023

**Published:** 2021-07-12

**Authors:** Lindsay Woodford, Lauren Bussey

**Affiliations:** ^1^Department of Health and Social Sciences, University of the West of England, Bristol, United Kingdom; ^2^Department of Psychology, Teesside University, Middlesbrough, United Kingdom

**Keywords:** Coronavirus, COVID-19, athlete wellbeing, mental health, photo elicitation, reflexive thematic analysis

## Abstract

Countries all over the globe have implemented mandatory social distancing measures in an attempt to suppress and control the spread of the Coronavirus disease (COVID-19). This enforced period of isolation, disruption to normal training routines and competition cancellation, could be having an adverse effect on the mental health and wellbeing of athletes. This study sought to explore the perceived impact of the COVID-19 social distancing measures on athlete wellbeing. Fourteen elite athletes who were unable to train or compete due to government imposed lockdown measures were recruited to participate in this qualitative study. Utilising the photo elicitation method, participants were asked to take a series of photographs that represented their experiences as athletes living in lockdown. These photographs were used to guide discussions in follow up unstructured interviews. Reflexive inductive thematic analysis identified three main themes that captured athletes’ experience of social distancing measures and the implications for their wellbeing: (1) threats to wellbeing; (2) adapting routines and maintaining motivation; and (3) reflecting on participation in competitive elite sport. The initial sudden loss of sport in the athlete’s lives posed a threat to their wellbeing, but over the duration of the lockdown period the athletes developed numerous strategies to protect their wellbeing. Furthermore, their time away from sport encouraged them to reflect on their athletic identity and to make life changes that would protect their wellbeing during the rest of the lockdown period and when they returned to sport. A number of immediate practical recommendations are offered for athlete support personnel working with athletes during the crisis, these include developing self-care strategies and social networks, adapting routines, setting new goals and encouraging the pursuit of dual-careers. Future research is encouraged to investigate how practitioners can deliver effective psychological support through tele-consulting, and to consider whether their support is best focused on therapeutic counselling or mental skills training during the pandemic.

## Introduction

On the 11th March 2020 the World Health Organisation (WHO) declared the coronavirus disease 2019 (COVID-19) outbreak as a pandemic. The situation is evolving rapidly with global case counts currently just over 122 million and deaths rates over 2.7 million worldwide on March 22nd, 2021 (World Health Organization, 2021). Whilst ongoing clinical trials and investigations aim to further our knowledge about the virus, its origin, and how it affects humans countries all over the globe have implemented mandatory social distancing measures in an attempt to suppress and control the spread of the virus. These strategies include; lockdowns, stay-at-home orders, curfews, non-essential business closures, bans on mass gatherings, school and university closures, travel restrictions and bans, remote working and quarantine of exposed people/travellers (World Health Organization, 2021).

Studies exploring the psychological implications of previous epidemics and pandemics have found social distancing measures to be particularly detrimental to psychological health (Taylor, 2019), with symptoms such as post-traumatic stress exacerbated by infection fears, longer quarantine duration, frustration, boredom, inadequate supplies, inadequate information and financial loss (Brooks et al., 2020). Similar findings have been identified in the COVID-19 pandemic, such as an association between negative changes in health behaviours (physical activity, sleep, alcohol and tobacco) and increased psychological distress (depression, anxiety and stress) (Stanton et al., 2020; Duncan et al., 2020), as well as increased psychological morbidity in those who identified as being at risk of contracting the virus (Jia et al., 2020).

There is an emerging evidence base that focuses on exploring the psychological implications of the COVID-19 pandemic on athletes, in response to the suggestion that athletes experience additional mental health risk factors compared to the non-athletic population (Schinke et al., 2018). Athletes have expressed substantial grief and frustration in response to this enforced period of isolation, disruption to normal training routines and competition cancellation (Pillay et al., 2020; Toresdahl and Asif, 2020; Gupta and McCarthy, 2021). These preliminary findings suggest that some pandemic mitigation strategies such as lockdowns, could be having an adverse effect on the mental health and wellbeing of athletes (Mann et al., 2020).

According to the World Health Organisation (World Health Organization, 2004), mental health is defined as “a state of well-being in which the individual realises his or her own abilities, can cope with the normal stresses of life, can work productively and fruitfully, and is able to make a contribution to his or her community” (p 1). Whilst mental health is a major resource for athletes in relation to their performance and development in sport, high rates of psychological distress and disturbance are reported among athletes (Markser, 2011) which may impair performance and wellbeing, according to the International Olympic Committee consensus statement on mental health (Reardon et al., 2019). Research suggests that the prevalence of mental health symptoms and disorders in male elite athletes in team sports (cricket, football, handball, ice hockey and rugby) varies from 5% for burnout and alcohol abuse, to nearly 45% for anxiety and depression (Nixdorf et al., 2013; Gulliver et al., 2014; Beable et al., 2017; Brown et al., 2017; Gouttebarge et al., 2017a, b; Schuring et al., 2017; Drew et al., 2018; Kiliç et al., 2018). In female athletes, mental health disorders range from 10% to 25% for depression and eating disorders in particular (Greenleaf et al., 2009; Proctor and Boan-Lenzo, 2010; Brand et al., 2013; Wolanin et al., 2016). There is also evidence to suggest that occurrence of such mental health disorders amongst elite athletes are associated with higher incidence of severe injuries, surgeries, recent adverse life events, a higher level of career dissatisfaction and a lower level of social support (Gouttebarge et al., 2017b).

Considering the increasing number of mental health problems being reported in the athletic population, the challenges that the pandemic presents to them, and recent research identifying the adverse effect of social distancing measures on mental health, athletes could be considered an at-risk group during the current COVID-19 pandemic. Indeed, this is reflected in recent findings that show the COVID-19 lockdown period significantly affected athletes’ physical and mental health. Specifically, physical deconditioning, worsening nutrition, uncertainty on return to sport, and disruptions to social interaction, training, and sleep patterns (Pillay et al., 2020) were associated with increased depression, anxiety and stress symptoms (Facer-Childs et al., 2021). Furthermore, in a recent study exploring adversity and resilience in athletes during COVID-19, athletes reported tangible losses with regards to their sport, support networks and athletic identity, and a sense of incongruence as a result of the dissonance between the usual structured, goal directed environment they were accustomed to, and the relatively aimless lockdown scenario presented by COVID-19 (Gupta and McCarthy, 2021). The incongruence experienced due to the lack of agency during the pandemic, resulted in psychological distress, ruminations, negative emotions and loss of motivation. Whilst the authors acknowledged the role resilience is playing in mobilising coping methods to protect athletes from these adversities, it could not be inferred that the athletes had positively adapted to the stressors as they are still living in a pandemic. Although the overwhelming evidence suggests that athletes have experienced negative physical and psychological effects of the COVID-19 pandemic, Şenışık et al. (2020) reported that the mental health status of athletes was better than non-athletes, attributing this difference to the potential protective effects of physical activity that were still experienced during their pause in sport participation.

Whilst the literature reviewed has furthered our understanding of the impact of social distancing measures on athlete mental health, the current research will utilise the concept of wellbeing as a framework for examining athlete’s experiences. Giles et al. (2020) argued that whilst athletes are by definition sport performers, first and foremost they are people whose physical, mental and social health is reflected through their wellbeing and ill-being. A consideration of athletes’ holistic health is fundamental to their identity as performers and people, and their participation in sport can contribute to or detract from their wellbeing. It is argued that the exploration of wellbeing in relation to athletes’ experiences of social distancing will provide a broader framework for investigation than mental health alone. Indeed, recent recommendations from a consensus statement on improving the mental health of high-performance athletes (Henriksen et al., 2019), identified various propositions to inspire researchers and support elite sport organisations to investigate and enhance mental health in athletes. Of particular relevance to the current study was their recommendation to better define mental health in sport as being more than the absence of mental illness, separate from performance, contextualised and acknowledging the full range of human emotions that are experienced.

Throughout sport psychology literature, researchers have found the construct of wellbeing to be both multi-faceted and complex. Lundqvist (2011) argues that “wellbeing is treated as an unspecific variable, inconsistently defined and assessed using a variety of theoretically questionable indicators” (p. 118). For the purpose of this research, the concept of wellbeing will be based on a review of existing measures of wellbeing in sport performers by Giles et al. (2020), who recommends an integrated measure of wellbeing that includes emotional, mental, social and physical components of wellbeing.

According to Stambulova et al. (2020), COVID-19 can be interpreted as a career transition barrier that interferes with athlete development and career progression. In the recent update of The International Society of Sport Psychology Position Stand on Athletes’ Career Development and Transitions (Stambulova et al., 2020), “career excellence” is defined as “an athlete’s ability to sustain a healthy, successful, and long-lasting career in sport and life” and not as “a destination to reach, but more a journey to, or process of, striving for it, in which athletes might need support” (p. 14). If we consider COVID-19 as a career development barrier from a holistic developmental perspective (Wylleman, 2019), it suggests substantial changes to athletes athletic development (e.g., closed sports facilities, postponed and cancelled competitions), psychological health (e.g., threatened athletic identity), psychosocial aspects (e.g., social isolation, worries about significant others), academic-vocational responsibilities (e.g., change in focus to education or work), financial concerns (e.g., funding cuts) and changing legal developments (e.g., restrictions on movement). Indeed, Gupta and McCarthy (2021) suggested that if social isolation is conceptualised as an abrupt diversion in an athlete’s career, it could trigger dissociations from their athletic identity and negatively impact wellbeing. Athletes often have a strong sense of identity that is derived from their participation in sport (Sparkes, 1998) and when this sporting platform is removed through injury or retirement, they lose access to vital resources and support networks which can result in threats to their wellbeing (Jewett et al., 2019). Preliminary findings suggest that the COVID-19 pandemic and lockdowns have presented athletes with a similar experience to that of forced retirement (Jewett et al., 2019; Park et al., 2013) and recommendations to support athletes using career transition and/or chronic injury psychological rehabilitation research as an intervention has been advocated (Gupta and McCarthy, 2021).

The current pandemic has presented sport and exercise psychologist consultants with challenges and opportunities with how best to support athlete’s sport performance and wellbeing. A recent editorial by Schinke et al. (2020) reviewed the challenges encountered by athletes such as social isolation, career disruption, qualification process uncertainty and unconventional and limited access to training facilities and training partners. Underpinning these issues was the need to help maintain the health and wellbeing of athletes in their pursuit of performance excellence during this extraordinary time. Schinke et al. (2020) further reflected on their collective work with aspiring Olympic athletes across Asia, Europe and North America and suggested strategies to support athletes that encouraged openness to express their feelings and facilitate problem solving, identification of gaps in their development, and the setting of new goals. Mascret (2020) reported that confinement during the COVID-19 pandemic encouraged athletes to modify their achievement goals; self-approach goals (improving oneself) and self-avoidance goals (avoiding regression). Specifically, they identified that self-approach goals decreased, and self-avoidance goals increased, suggesting that during confinement athletes were more focused on avoiding performance decrements than enhancing it.

In order to support athletes during this continuing global health crisis, further research is needed to investigate how social distancing measures are affecting athlete wellbeing. Given that isolation periods are detrimental to wellbeing (Taylor, 2019), and that elite athletes are at greater risk of mental health disorders (Reardon et al., 2019), together, the impact of the COVID-19 social distancing measures on elite athletes could be profound. It is vital that exploratory work which seeks to understand the lived experiences of athletes is conducted, to contribute to a limited but growing evidence base, in order to provide a foundation for future intervention studies to support athlete wellbeing, and to help inform Sport and Exercise Psychologists recommendations/practice.

Research question

This study aimed to explore the perceived impact of the COVID-19 social distancing measures on athlete wellbeing. Specifically, the authors sought to investigate the emotional, mental, social and physical components of wellbeing that may have been affected by the lockdown measures.

## Materials and Methods

### Theoretical Underpinning

According to Mayan (2009), qualitative studies should have methodological coherence, which this study achieved by ensuring congruence between epistemological and ontological positions, theoretical perspective and the research question. This study adopts a realist ontological viewpoint which acknowledges there is an objective reality of the material world, and also takes a critical realist epistemological perspective, accepting multiple accounts of any phenomenon (Maxwell, 2012). As described in Maxwell (2012) “critical realists thus retain an ontological realism while accepting a form of epistemological constructivism and relativism (multiple versions of a phenomena can exist)” (p.5). By taking this stance, this research was able to explore how athletes perceived the impact of social distancing measures in the COVID-19 pandemic on their wellbeing.

This qualitative research collected data through combining the participant driven (open) photo elicitation method (Bates et al., 2017) with unstructured interviews, and was analysed using thematic analysis (Braun and Clarke, 2006). Conceptual coherence between theoretical and conceptual underpinnings of the research and the type of thematic analysis carried out is one of several proposed indicators of thematic analysis research quality (Braun and Clarke, 2020). In line with our aforementioned theoretical perspectives a reflexive approach to thematic analysis was taken, acknowledging and embracing researcher subjectivity as an analytic resource (Braun and Clarke, 2020). The choice to carry out an inductive thematic analysis was informed by the understanding that the analysis remains grounded in the data (Braun and Clarke, 2020). Furthermore, this form of analysis ultimately respects the participants’ unique experiences of the COVID-19 pandemic, and thus follows on from our previously stated ontological and epistemological position. To further ensure that the themes developed from the data are true to the participants’ overt understanding of their experiences (Braun et al., 2016), we employed semantic coding and did not aim to interpret any hidden or underlying meanings.

### Photo Elicitation Method

The rise in accessibility and advancement of visual technology and communication through social networking sites and smartphones means that a wealth of contemporary phenomena and experiences are captured visually (Bates et al., 2017). The photo elicitation method uses photographs within an interview setting in order to prompt emotional connections to memories and evoke more meaningful accounts (Hogan and Warren, 2012; Kunimoto, 2004). Bates et al. (2017) describe the following three variations to the photo elicitation method: (1) Participant-driven (open): participants choose to take any photo they feel is relevant to the phenomenon under exploration (2) Participant-driven (semi-structured): the researcher shares a set of questions with the participants and asks them to take photos that align with those (3) Researcher driven: the researcher provides the photos for the interview to stimulate discussion only.

The photo elicitation method has previously been implemented to explore elite runners’ disordered eating (Busanich et al., 2014), to capture the experience of Ironman competitors (Wakefield and Watt, 2012), and in a similar vein, creative collages using images has also been used to understand young athletes future career aspirations (Ronkainen and Ryba, 2018). The current study utilised the participant-driven (open) format as it was considered most appropriate to address the exploratory nature of the research question. By encouraging the production of participant-driven photographs the researcher can attempt to understand the experiences of the participants (including emotions and ideas) rather than imposing their own framework or preconceived ideas on the topic (Holloway and Galvin, 2017). The purpose of the images, therefore, is to create a dialogue and to introduce new dimensions that had not been considered by the researcher, and in this respect is easily facilitated by the accompanying unstructured interviews.

### Recruitment

As employed in previous qualitative work around athletes’ experiences (e.g., Kirby et al., 2011), purposeful sampling was utilised to recruit athletes residing in countries enforcing strict lockdown measures which prevented them from leaving their house to train and compete in their sport. Participants were recruited through the researcher’s network in elite sport and sustained through snowball sampling (Patton, 2002). However, none of the participants were known to the researchers so there was no potential for coercion. Contact was made to prospective participants through ethically approved invitations via email or through private messaging on public social media sites such as Twitter. The exclusion criteria were displayed in study invitations. Participants were unable to participate if they: were under the age of 18, had any existing clinical mental health problems, had contracted COVID-19, were recreational athletes, or resided in countries who did not have to adhere to lockdown measures. A heterogeneous sample of athletes were recruited to ensure that the research captured a wide range of athlete’s experiences from team and individual sports.

### Participants

A total of 14 elite athletes (eight White women, one Indian woman and five White men) representing 10 sports who were unable to train or compete due to government imposed lockdown measures participated in this study. Whilst the severity of the lockdown measures differed across the countries none of the athletes in the study were allowed to leave their homes to participate in organised sport.

In order to better define the sample, the athletic level of the participants was classified based on their highest standard of performance, their success at that level and the amount of experience they had gained at that level. Adopting the four-tiered classification system set out by Swann et al. (2015) participants were categorised as semi-elite; competitive elite; successful elite and world class elite. *Semi-elite athletes* participated below the highest level possible in their sport; *competitive-elite athletes* regularly competed at the highest level but had not achieved success; *successful-elite athletes* competed at the highest level and had experienced some success; *world-class elite athletes* competed and experienced sustained success at the highest level (e.g., Winning Olympic medals in consecutive games or major competitive victories over a number of seasons). To illustrate how participants met criteria for this research, pertinent demographic characteristics are provided in Table 1.

**TABLE 1:** Participant demographic information.

Participant	Age	Sport	Elite level	Country of residence
Zoe	18	Athletics	Competitive-elite	South Africa
Dave	59	Biathle	Successful-elite	South Africa
Jess	21	Hockey	Semi-elite	United Kingdom
Kate	46	Fencing	Successful-elite	South Africa
Rob	51	Biathle	Successful-elite	South Africa
Hannah	18	Biathle	Competitive-elite	South Africa
Charlie	29	Rugby	Successful-elite	France
Olivia	18	Biathle	Competitive-elite	South Africa
Rachel	42	Powerlifting	Successful-elite	United Kingdom
Luke	30	Cricket	Successful-elite	Zimbabwe
Ben	22	Athletics	Competitive-elite	United Kingdom
Annika	48	Cross country	Successful-elite	South Africa
Sarah	18	Gymnastics	Successful-elite	South Africa
Amelia	25	Bodybuilding	Competitive-elite	United Kingdom

### Procedure

Upon recruitment, participants were informed of the study aims and procedure, and then provided their consent to participate. Participants were instructed to take any number of photographs they felt represented their experience of the COVID-19 pandemic as an athlete, using any available technology (e.g., iPads, smart phones, or digital camera). They were informed that the photos should not contain any people or easily identifiable places and reminded that they were a conduit to aid discussions and would not be part of the data analysis; only the associated narratives would serve as the data set (as recommended by Bates et al., 2017). Participants were contacted again 2 weeks later to organise an online interview, and to submit via email any photographs they wished to discuss in the interview.

All interviews took place between April and May 2020 and were conducted virtually via Microsoft Teams due to the geographical spread of participants across the globe and lockdown regulations in place at the time of data collection. This virtual platform also provided a secure encrypted connection to ensure confidentiality. The online interviews were held in private rooms and the participants were informed that if they felt any discomfort discussing their experiences, they could terminate it without any questions asked.

Each interview was audio recorded and lasted between 45 and 60 min. In accordance with the participant driven (open) photo elicitation method, there were no pre-determined interview questions. The researcher began each interview asking, “Please could you talk through these photos and explain why you chose them to discuss today?” and asked follow up questions when appropriate. In this sense, the photos selected and discussed by the participant framed the interview process, leaving the control and power of the interview process as much as possible with the participant (Bates et al., 2017).

To protect anonymity participants were assigned pseudonyms and any identifying information was redacted from transcripts (Braun and Clarke, 2013) and interview data were stored in a password protected computer. Participants were debriefed following the interviews and given an information document that signposted to support resources in the event that any of the discussions had prompted any adverse psychological or emotional reactions.

### Data Analysis

Due to the exploratory nature of this novel research, reflexive inductive thematic analysis (Braun and Clarke, 2019, 2020) was used to analyse the data and the analysis process was informed by Braun and Clarke (2006) six phases of analysis. In order to achieve data familiarity, the researcher first transcribed interviews verbatim using NVivo software and engaged in repeated reading of transcripts and re-listening to the data, helping to move the analysis beyond a focus on the most obvious meanings. In the second stage of the analysis, all data relevant to the research question were coded. This process identified the first patterns in the data by grouping similar data segments at the semantic level. Similar codes were then clustered together to create a visual map of key patterns in the data, these existed in a hierarchical structure containing overarching themes, themes and sub-themes. The themes were reviewed to ensure they fitted with the coded data and the overall data set, and that they each had a clear organising concept. At this stage, the second author reviewed the themes to ensure they were substantially supported by data extracts, and contributed to answering the research question. The final themes and subthemes were then clearly defined and summarised to provide conceptual clarity and a road map for the final report.

### Methodological Rigor

In alignment with the aforementioned conceptual underpinnings of the research, the authors engaged in careful design thinking to conduct reflexive thematic analysis research with methodological integrity (Braun and Clarke, 2021) and to ensure the validity of findings (Morse et al., 2002). For example, the lead author engaged in regular journaling, reflecting on the process to ensure an in-depth engagement with the data resulting in a meaningful analysis, as recommended by Braun and Clarke (2021). Furthermore, the lead author carried out the initial coding process which was reviewed by the second author, this process was repeated at the initial theme development stage, as per Braun and Clarke (2021) recommendations for best practise in reflexive thematic analysis. Credibility was ensured by giving participants the opportunity to check transcripts for accuracy, and to engage in a process of member reflections with the lead researcher (Tracy, 2010) to explore any gaps in the results and to share any similarities in the interpretations of the findings (Schinke et al., 2013). The analysis was also discussed and cross analysed at various stages by the lead researcher’s professional doctorate supervisory team to ensure worthy contribution to the topic and meaningful coherence (Tracy, 2010). As such, we feel that this research is timely and that the findings contribute unique insights regarding athlete wellbeing throughout the COVID-19 pandemic, afforded by our use of the photo elicitation method that is seldom used in sport and exercise literature. Finally, the analytic write up contains data excerpts and analytical commentary to provide strong evidence of patterning across the data (Braun and Clarke, 2013), which we hope resonates with other members of the elite sporting community also affected by COVID-19 mitigation strategies.

### Ethics Statement

Ethical approval for this study was obtained from the Research Ethics Committee School of Social Sciences, Humanities, and Law at the University of Teesside.

## Results

Following the participant driven (open) photo elicitation method, participants provided the researcher with photographs they had taken which documented their experience as an athlete living in lockdown, due to the global COVID-19 pandemic. Though the images were important to elicit discussion, they were not part of the analysis. Reflexive, inductive thematic analysis (Braun and Clarke, 2006) was applied to the participant’s discussions of the images and identified three main themes which highlight their experiences of lockdown as athletes, these were (1) threats to wellbeing; (2) adapting routines and maintaining motivation; and (3) reflecting on participation in competitive elite sport.

### Theme 1: Threats to Wellbeing

This theme is defined by athletes’ discussions that focused on how the lockdown and social distancing measures negatively affected their wellbeing. Athletes talked about how existing worries were exacerbated by lockdown, including maintaining a balanced diet, body image, mental health and injury recovery (subtheme 1), and how limited training opportunities and reduced access to outdoor space threatened their wellbeing (subtheme 2).

#### Subtheme 1: Lockdown Impedes Basic Athlete Needs

Following the initial shock of the COVID-19 crisis and its ramifications for participation in sport, the continued lockdown period posed a threat to wellbeing for those athletes who were already coping with some form of adversity in their lives. Pertinent to the pandemic, Amelia spoke of the stress she experienced when she could not buy the food she needed to maintain her physique because of the panic buying in supermarkets:

*So not being able to access my usual food in lockdown was really stressful at first, in my head I was thinking oh my goodness if I don’t have 120grams of protein today I am going to lose all my gains. But if I took a step back and thought logically I knew I was not going to lose two years’ worth of muscle in the space of a week because I couldn’t get my hands on the food I needed… but at the time I thought my whole world had come crashing down, first they cancel my competitions and now I can’t maintain my nutrition or physique because I can’t eat the food I need to eat, I just can’t cope with this. (Amelia, 25, body building, United Kingdom).*

Jess also expressed physique concerns as she described noticing physical changes to her body due to the lack of training which translated into poorer mental wellbeing. The selfie she took in the bathroom mirror illustrated how her pre-existing body image struggles had been amplified during the lockdown:

*In terms of the other things like my hair and my skin, and even my nails they’re not growing the same way. I just don’t feel that nice and that’s when I started getting down which is a bit concerning. I have tried not to look in the mirror to be honest, I just try not to overthink it. I haven’t been on the scales, because I just actually point-blank refuse at this point. I know it’s the physical activity, the lack of it makes me feel disgusting. But yeah, it’s horrible, I’m just managing it, I really just try not to think about it. I’ve always struggled with kind of weight. And I always knew that this period, I said to myself this is gonna be hard, you’re going to gain some weight. As soon as the restrictions are lifted. I’m really motivated to get rid of it, already. (Jess, 21, hockey, United Kingdom).*

For many, participation in sport and exercise was used as a way to de-stress and to channel both physical and mental energy in a positive activity. This is exemplified by Rachel, who shared a photograph of her empty training diary and described how competing in sport had been a way of managing her attention deficit hyperactivity disorder (ADHD) and maintaining her wellbeing prior to lockdown:

*I always needed the physical stimulus of sport but didn’t know why, it wasn’t until after the diagnosis of ADHD and then only last year when I was doing some work with my counsellor and I said I feel guilty about training because I haven’t started working and I have to collect my son and my days are really short. She said have you thought that maybe you need it in order to function and that was a massive light bulb moment for me. I didn’t realise, she was right, she was spot on. I need my sport to manage my mental health. (Rachel, 42 powerlifting, United Kingdom).*

Referring to a photograph of him participating in rehabilitation training at home, Charlie described how the psychological implications of injury recovery were more difficult to cope with during lockdown. Charlie described how not being able to see friends, family, or his teammates exacerbated feelings of social isolation during the lockdown:

*It’s been tough having an injury in lockdown, usually I would use this time off to spend time with friends and family which I don’t usually get time to do, but with lockdown you can’t. Sat at home being isolated trying to find ways to connect with people, but it’s not the same as face to face and I have found that difficult. Being injured can be quite isolating, you are away from the rest of the squad, you don’t get those social interactions at the club that you really enjoy, and you feel like you are away from the group and so you go and see family and friends and to not be able to do that is pretty tough. (Charlie, 29, Rugby, France).*

Rob also echoed this idea that being isolated from fellow athletes throughout lockdown posed a threat to wellbeing, as he described relying on team training as an opportunity to offer and receive social support, which he and his family miss:

*As a family we miss the social interaction, my wife has a strong bond with a lot of the women in the training group and we all miss that as much as we miss the running itself, we are a bunch of good friends who support each other through difficult times. We can talk to each other on the phone but it is that actual physical interaction with a person that we miss. (Rob, 51, biathle, South Africa).*

Overall, the athletes discussed how existing stressors were exacerbated by the pandemic restrictions, which they seemed to have negatively affected their wellbeing.

#### Subtheme 2: Lockdown Stunts Athletic Development

This subtheme contributes to the overarching theme as many of the athletes shared how the lockdown had stunted their athletic development which they felt negatively affected their wellbeing. In response to their reduced access to training facilities the athletes made attempts to adapt equipment they had at home. Amelia shared a series of photographs of the weightlifting equipment she and her Dad had built together during lockdown. She described how her reduced financial income paired with price increases on home training equipment, encouraged her to build her own training kit at home with her Dad. According to Amelia, this had a positive effect on their father-daughter relationship and her wellbeing:

*That photo is of a drill on a bench. So being on furlough I couldn’t afford to buy any decent kit. So, my Dad built me a squat rack, we built that together, so it was a bonding thing, so I could work out. I just couldn’t afford anything, it felt like everywhere shot up their prices when lockdown happened so the only way around it was to build it myself. My Dad has been so supportive of me and he’s always pushed my athletic side and thrown money at all the sports I played as a kid, but nothing ever really stuck so he’s seen my passion for body building and I’ve made it me, and he’s been so supportive and anything he could do to help he was happy too, let’s build the equipment you can’t buy and make weights if we have too he told me, which was really nice. (Amelia, 25, body building, United Kingdom).*

Some athletes turned to social media to share their home training efforts, in order to feel connected with others in their sport. For example, Sarah shared images of herself training at home on Instagram with fellow gymnasts, which helped her feel part of the gymnastics community despite the lockdown restrictions:

*As you can see in the photo of me doing a handstand on a wheelie bin, I have been posting lots on Instagram which helps you feel like you are in the world, that I am in the gymnastics environment, so it does sort of help. It keeps me motivated as I can see that everyone else is training at home and my friends call me and we are there for each other. (Sarah, 18, gymnastics, South Africa).*

Although Sarah found some solace in sharing her training online, other athletes found such updates to be damaging to their wellbeing, triggering downward social comparisons of body image and their own ability to cope with lockdown. Jess and Olivia described feeling pressured to be proactive and accomplish goals during the lockdown period which had a negative impact on their wellbeing:

*So, some girls on my team are skinnier than me, or better than me and I have never gone I wish I was like that, it has never impacted me because I’ve always had that self confidence that I am who I am and, to an extent I really do like who I am and I’m happy with how I am and what I’m doing and my achievements. But now, for some reason, it has hit me like should I be doing that? Why am I not doing that? How can I do that? Or why are people not including me in that? I’ve been noticing the most intricate things, and I’m thinking to myself, you’re never normally like this, you shouldn’t even be focusing on it. (Jess, 21, hockey, United Kingdom).*

*All over social media there is this thing that we need to use this time to get motivated and be so much better when we come out of the other side. But for some people they need a break, to slow down and relax for a while because all this bettering yourself might not be what you need right now and I think a lot of people are being pressured to think that they have to get better, to use this time productively and they beat themselves up if they don’t, it is a really big thing right now… they shouldn’t feel pressured to come out of the other side so much better than when they came in. (Olivia, 18, biathle, South Africa).*

By immersing themselves in their respective virtual sporting communities, the athletes not only compared their adaptation to lockdown life with other athletes online, but they also noticed inequalities with regards to access to equipment. Ben expressed feeling disadvantaged due to his lack of home training equipment:

*A lot of people are putting stuff on social media who have managed to get access to equipment, and you wonder well if they’ve got that then what advantage have they got over me? (Ben, 22, athletics, United Kingdom).*

Similarly, Olivia described feelings of animosity towards fellow athletes who could access more equipment than her, but also felt uplifted and inspired by some of the stories shared:

*But there’s definitely been jealousy on social media with people who have access to more than I do at the moment, but it is also really cool to see people in similar situations who are fighting through this and are coming together to get through this. (Olivia, 18, biathle, South Africa).*

Underpinning the above conversations regarding athletic development during lockdown, there was a clear, common effort amongst the athletes to find alternative ways of training at home, although this did not always alleviate feelings of social isolation. Sarah a young gymnast, captured her experience of loneliness when training at home in an image of a sunset:

*This photo of the wall with a sunset behind reminds me that my teammates are not with me and you are by yourself you are alone, it’s different when you are in the gym there are about 100 people in there training with you and supporting you, but you don’t have that being at home. It is an empty open space; it makes me sad. (Sarah, gymnastics, 18, South Africa).*

Training from home not only heightened feelings of loneliness, but also blurred the boundaries between athletes’ multiple identities which Rachel found difficult to manage:

*I need to have my places that I go too to do stuff, like the gym is where I train and eat, I go to uni to be quiet and have space and my son and dog can’t come. I need to have those places to function, but I don’t have those places and now my life isn’t functioning. (Rachel, 42, powerlifting, United Kingdom).*

Rachel went on to explain how training from home was particularly challenging, but she was determined to make it work as her wellbeing depended on it:

*This is a photo of my makeshift home gym. So yesterday I had the first training session in it, I built it last week, it was ready to go but there was so much other stuff in there and I thought ugh I can’t. I had to move the fridge freezer to get the plates on the bar, there’s a lawn mower so I can’t get into the cage I have to move it. It’s really dusty in there so the first time I thought argh this isn’t working. So, the first lift when I had to breath in and brace, I got a mouthful of dust and wasps flying around my head and the dog was under my feet. I thought this is dangerous I can’t do this I think I just did ten squats and then I thought I can’t do this, I can’t use this gym, I can’t lift safely and I can’t get into the space into my head that I needed to with all that shit in there. (Rachel, 42, powerlifting, United Kingdom).*

Despite the differences in lockdown restrictions between countries, many athletes acknowledged the instrumental role that spending time outside had on their wellbeing. Luke reflected on a photograph of him doing circuit training in his small garden and discussed how the lockdown restrictions limited his opportunity to exercise outside, for which he relies on for a natural biochemical mood boost:

*We are all athletes and love being outside doing something that engages the endorphins, so to not be able to get the blood flowing and those positive vibes has been hard. (Luke, 30, cricket, Zimbabwe).*

Similarly, Amelia described how her daily walks by the canal were an uplifting experience helping to relieve the pressure of living in close quarters with her family during lockdown:

*So that is a photo of my caterpillar boots, throughout lockdown every morning I would stick them on and take myself out for an hour or two hours and go walking up by the canal, I am lucky where I live there are lots of beautiful places round here to walk…it was my meditation, those boots have done some mileage over the last year… During the lockdown my Dad was also furloughed from his job, so we were stuck in the house together, we are very close, but like any family you rub against each other eventually, so my walking was my time, it was my time to get out of the house and call my friends or stick my headphones in and listen to music or a podcast, it was my time to myself which I didn’t have in the house. (Amelia, 25, Body building, United Kingdom).*

For some athletes like Rob, the importance of access to outdoor space became even more apparent as he reflected on his friends’ efforts to train with limited indoor space:

*Friends who stay in an apartment block have had to get permission from everyone in their apartment block to run up the flights of steps, we are so fortunate with the size of our garden and the treadmill. Their level of frustration is a lot higher than ours, they just want to be able to get out on a road and be less restricted. (Rob, 51, biathle, South Africa).*

In this subtheme, athletes discussed how the lockdown stunted their athletic development as a result of the lack of access to training equipment, social support and limited space, which negatively impacted their wellbeing. Various strategies were utilised as coping methods to facilitate their athletic development and to protect their wellbeing, such as building their own equipment, connecting with teammates through social media, and participating in exercise outside where possible to boost mood and maintain healthy wellbeing.

### Theme 2: Adapting Routines and Maintaining Motivation

In this theme, the athletes mobilised coping resources such as adapting their routines and changing their motivational focus to manage the diminishing psychological wellbeing they experienced due to their lack of agency during the lockdown. They reflected on how before the pandemic their lives were structured and goal driven, but the lockdown scenario had created an aimless existence which threatened their wellbeing.

#### Subtheme 1: Replacing Old Routines With New Ones

A key aspect which some athletes felt they lost in lockdown was their training routine, which for many provided a structure to their day. For Rachel, the lack of routine had deleterious implications for multiple aspects of her wellbeing, she discussed this as she shared a photograph of a bottle of gin and a kitchen cupboard filled with junk food:

*I had nothing to do, literally nothing to get up for and I lost momentum. I went to bed later and later every night and then got up later every day and within a week and half I couldn’t sleep, I was staying up late watching Netflix and then going to bed late, having crazy dreams, probably because of the alcohol but also because everything was just weird. Then waking up really late and the whole day I felt like I was on the back foot. Nothing was getting done it was one long thing of sleeping and staying up late and not getting much done at all. My routine went out the window and I felt awful. Now I have one it feels better. (Rachel, 42, powerlifting, United Kingdom).*

Similar to Rachel, Ben also shared the challenges of having so much spare time and how adapting his routine had helped ease the boredom and frustration:

*It was quite hard to adapt in the first couple of weeks and get into a routine and now I have that routine of I wake up and do a little circuit or something in the morning and then do some uni work and then go out training in the evening and now I have that routine it gives me a good structure and gets me through the days. It is quite good to have that routine or you sit around doing nothing all day. (Ben, 22, athletics, United Kingdom).*

Some athletes involved their family members in their new routines, which had a positive impact on their wellbeing. For example, Sarah spoke of how she had coped with the decreased feedback from her gymnastic coaches during the lockdown, by becoming more autonomous with regards to her training and her parents taking on coaching roles:

*In this picture my Mum and sister are holding onto the bar, we normally hang our washing on it, but it’s not that strong and I have been working on it to improve my grip. I have grown a lot because I used to rely on my coach to help me do stuff, but now they are no longer there I have to motivate myself and do it all by myself, so I think I am more independent. My parents have learnt a lot in the process as I have had to teach them how to spot. I am so used to my coach knowing what to do so I had to teach them. My family has been my biggest support during lockdown. (Sarah, 18, gymnastics, South Africa).*

However, it was not only about setting new athletic routines that was protective for wellbeing, the inclusion of new non sport related hobbies were equally effective at maintaining wellbeing. Olivia described how taking up photography helped pass the time, but also acted as an outlet for expressing her feelings during lockdown:

*During the lockdown and having all this free time I have gotten into photography right. So, I have been playing with the camera and me peeking through the fence in that photo I was trying to get the same feeling as being isolated from other people and that was the best thing I could do, being isolated from the outside, not being able to leave and not being able to have the freedom of choice and that sort of stuff. (Olivia, 18, biathle, South Africa).*

Luke took a photograph of his PlayStation console to explain how his new routine involved playing online video games with his teammates during the lockdown. He felt this helped him to stay socially connected with fellow cricketers and also filled some of the competitive void he was missing from playing sport:

*So Call of Duty, I am a very competitive person and I like to do things that involve competition and so obviously being locked up there are not many ways that we can find to do some competitive things, so having some online gaming has been entertaining and kept me busy and it’s been nice because I’ve been playing with a lot of other friends, which is pretty cool, there are a lot of international cricketers that play so there’s a lot of banter and stuff on the game which is quite cool. (Luke, 30, cricket, Zimbabwe).*

For others like Charlie, the simple act of setting small, achievable, non-sport related goal each day was a useful strategy to protect his wellbeing:

*I sent you a photo of a big hedge I trimmed, that was a whole day’s work, it wasn’t something I wanted to do, it just had to be done, it is about setting goals and that was my goal for the day. Being a sportsman, you are so used to having goals, you have games at the weekend and that is your goal all week, so doing things like that has helped me see that it is not just another day, it’s the hedge day or the grass day or painting and it has been really important, it’s really helped me. (Charlie, rugby, 29, France).*

Overall, this subtheme encapsulates how athletes replaced their usual strict training routines with new activities that enabled social connection and emotional expression, helping to support their wellbeing during the pandemic.

#### Subtheme 2: Motivation for Training in Lockdown

Whilst many athletes adapted to the lockdown by creating new routines, many of the athletes discussed the challenges of motivating themselves to train for their sport during lockdown, as they were unable to access training equipment and they were uncertain when competitions would resume:

*I didn’t know why to train in quarantine, I had no big events coming up, I thought why should I train? (Olivia, 18, biathle, South Africa).*

*It’s been quite lonely, and my motivation has been quite low. It has been in peaks and troughs, sometimes I am really motivated and have really good days and some days I do nothing at all. Keeping my motivation has been harder than anything else, just keeping my goals in mind despite the situation that is going on (Jess, 21, hockey, United Kingdom).*

Adjusting goals from ego orientated to task orientated in light of the competition postponements and cancellations helped maintain their motivation to train despite the lack of agency in their sport:

*You don’t have the coach forcing you to pitch up at training or your Mum forcing you to get out of bed to take you to training, you have to do it yourself and find the reasons behind why you are doing what you are doing. (Olivia, 18, biathle, South Africa).*

*My training diary is a good way of keeping track of what I am doing and that helps motivate me. Normally my sessions would be 2x200m and I’d know exactly what times I need to hit, but now I don’t really know what a good time is to run across a football pitch in, or from one lamp post to the next, none of these things mean anything to me at the moment, it is all relevant to just me. (Ben, 22, athletics, United Kingdom).*

In addition to changing their motivational focus to one of self-improvement in their sport, Hannah described how she was exercising to maintain her wellbeing:

*As you can see from this photo of our pool it’s not very big, but I swim in the morning, it is my favourite part of the day even though it is quite cold. It is not difficult it is not like training to keep fit, it is really fun, every time I get out of the pool, I feel refreshed. It is a good start to my day. It lifts my mood. (Hannah, 18, biathle, South Africa).*

It seems that some athletes relied upon external motivations from family members to continue to train during this period of uncertainty in their athletic career:

*I just try to get by week by week and motivate myself by; oh, look my Dad is running today I should run as well. So, my family plays a big role in motivating me. (Zoe, 18, athletics, South Africa).*

*With the four of us in our family running, if the kids aren’t motivated my wife will motivate them to get on the treadmill or do a couple of laps around the garden. The community within the family is very motivational. (Rob, 51, biathle, South Africa).*

Annika chose a photograph of her family running around the garden to illustrate how they had motivated each other through exercising as a group:

*We are doing a Park Run every Saturday; we have done four since lockdown and everyone must participate… we are running around our house, we have a big area where we can run, we are not allowed outside of our house… My son follows his time and he and his older brother compare their times, the rest of us we are just enjoying it. (Annika, 48, cross country, South Africa).*

Whilst some relied upon the people they lived with during lockdown for motivation, Ben was enjoying competing online against other runners in his local town by setting up routes to race on through the smartphone application Strava which he shared a photograph of:

*On Strava you can map your run and the whole athletics community can get involved and you can do segments to find out what other people have run, and you can get a crown for running the fastest time on that segment. There is a segment that runs from lamp post 77 to 82 in my town which used to belong to my brother in whatever time he did, he was the champion of that segment so I went out and did my run to try and get it and I did so it’s quite a good way to keep that competitive element of athletics by trying to race and become the fastest on different areas of road that are dotted around my town. Although you can’t race against anyone in close proximity. (Ben, 22, athletics, United Kingdom).*

In this subtheme, the athletes described relying upon family and friends to motivate and encourage them to train for their sport during lockdown, perhaps in replacement of teammates and coaches. Others however were motivated not by the pursuit of athletic development, but to protect their wellbeing during the lockdown, such as exercising outdoors to boost mood.

### Theme 3: Reflecting on Participation in Competitive Elite Sport

Towards the end of the first lockdown period the athletes began to reflect on their athletic career. It seems the time spent away from competitive sport encouraged them to consider its place in their lives. Some engaged in a cost-benefit analysis of their participation with regards to the impact it had on their wellbeing.

Dave began to reflect on the financial strain associated with competitive sport and questioned if the current pressure he felt to recover from his injury was outweighed by the rewards of a gold medal:

*I had planned to do the Provincial Biathle Champs and the Nationals, I probably won’t go overseas as it is expensive, and I now question its value. It has all been cancelled now and I have broken my elbow, it may never be straight again. I have a lot of pressure to get this arm straight. But the other side of this thing is why am I competing? Is winning and getting a gold medal what it is really cut out to be? (Dave, 59, biathle, South Africa).*

Reflecting on a photograph of his professional rugby kit, Charlie also commented on his changing view of the importance of competing:

*But now it’s like bloody hell we were really onto a good thing here and could have gone into the top league and had an amazing time, but that’s disappeared as well and the longer it has gone on you realise that its only one year, one season and seasons come and go and I’ve done 13 of them or something, and as much as winning and being at the top is great it doesn’t mean anything really, not when you really think about it, it’s just a moment in the sun isn’t it? (Charlie, 29, rugby, France).*

For some participants, lockdown restrictions gave them time to reflect on how damaging participation in elite sport could be to their mental and physical wellbeing, being rather thankful for the time out. When looking at a photograph of his race number, Rob shared his experiences of debilitating performance anxiety whilst competing and the relief he felt that this year’s competitions had been cancelled due to the pandemic:

*I honestly think it is a good thing not to be competing, on race day my emotions are a combination of nerves and excitement, I usually have to go to the bathroom 3-4 times on the morning of an event, my stomach works overtime. It is nothing to do with my diet I have tried every variant of breakfast and lunch and dinner, nothing helps, it is just my nerves, I have a toilet roll in my back pocket. I put exceptionally high expectations on myself. I think that there has been more benefit to not racing from the psychological point of view, less stress on me. (Rob, 51, biathle, South Africa).*

Like Rob, Luke also seemed to appreciate the time out provided by the pandemic, describing how before the lockdown he had continued to play hurt despite his injury, and that the lockdown had allowed him to take the time he needed to recover from his surgery and mentally prepare for his return to play:

*But this came at a good time for me as I actually tore my groin and my abdominal wall in January, but was still competing, I had an operation at the end of January so it’s been quite nice as I was actually supposed to come back to play but it has given me a longer recovery period, so it’s a kind of blessing in disguise as I can actually get my body back strong. I need to be in a good head space and have confidence in my body to be able to push it to its limits. It effects my performance a lot if I am not ready. (Luke, 30, cricket, Zimbabwe).*

Whilst some of the athletes used the pause in competition as time to reflect and recover in preparation for their return to sport, others like Jess reflected on the importance of alternative career paths. Jess spoke of her identity as a student-athlete and how having a dual career was protective for her wellbeing, giving her something else to focus on during lockdown. She shared a photograph of a pile of research papers to illustrate this:

*The photo of all the research papers is to show my identity as student now and that’s what I have been doing with the majority of my time. Being a student-athlete has been protective for my wellbeing, definitely, in terms of my identity it has shifted a little bit.* (Jess, 21, hockey, United Kingdom).

Charlie also considered how the time afforded by his injury during lockdown had forced him to think about what he was going to do after he retired from rugby, as he shared a photograph of an online apprenticeship programme he was studying:

*The fact that we are in lockdown it has given me a lot of free time to look at other things and it’s important thing for me to have another route outside of rugby. To make sure that when I come out whether it’s the next two years or ten years, I have something to fall back on and have credentials so I can do what I want to do outside of sport. Before the lockdown I was playing rugby five times a week and there wasn’t much time to look at stuff like this, at that point in time I was coming home from a long days training and was pretty tired and it was hard to switch into the mindset of studying. It has been nice to have it now as a flip from rugby. The thing that lockdown has done is show 100% that you do need these things in place especially as rugby or sports careers are not everlasting at some point you will have to go out and do some sort of other work and with my injury that is something else that showed that. (Charlie, 29, rugby, France).*

Overall, the lockdown highlighted the fragility of the athlete’s careers, and the extra time afforded by the cessation of their sport prompted them to explore alternative career paths that would help protect their wellbeing during subsequent periods away from sport.

## Discussion

This study was conducted in response to the growing number of athletes seeking sport psychology support during the pandemic, and the need to develop evidence-based interventions to inform best practice. Using a qualitative open photo elicitation method, this study explored the impact of the COVID-19 social distancing measures on athlete wellbeing. Three main themes; threats to wellbeing, adapting routines and maintaining motivation, and reflecting on participation in competitive elite sport, were developed through the process of thematic analysis, and the main findings are discussed below.

### The Loss of Sport Impacts Wellbeing

Overall, the athletes in this study provided narrative evidence for Giles et al’s. (2020) conceptualisation of wellbeing as an integrated measure of emotional, mental, social and physical health. They shared their feelings of loss, shock and sadness at the cancellation of their sporting events, and spoke of the threat to their physical and mental health as a result of the social isolation during the lockdown. The current research identified athletes’ feelings of loneliness from the lack of social interaction with their athletic peers and the negative impact this had on their wellbeing. Tomalski et al. (2019) similarly identified student-athletes as a unique population at a heightened risk for the development of mental health problems, coupled with a decreased willingness to seek help. It has been reported that periods of reduced physical activity compound feelings of isolation from teammates, distance athletes from the athletic community, allow less interaction with coaches and this lack of social support can cause emotional and psychological distress (Reardon et al., 2019).

Athletes also discussed how social media was used as a crux in order to immerse themselves in their sporting world, but which counterproductively encouraged downward social comparisons against fellow athletes who seemed to be coping better with lockdown, and highlighted inequalities regarding equipment accessibility which heightened return to play competition anxiety. Whilst not athlete specific, early research from Wuhan, China the first epicentre of the global pandemic has reported that excessive use of social media during lockdowns has led to an increase in mental health problems. The authors recommended that taking a social media break during the pandemic may promote wellbeing and mitigate the mental health repercussions of the pandemic (Zhong et al., 2021).

### Negotiating the COVID-19 Athletic Career Transition

The athletes identified numerous barriers to their athletic development and the maintenance of their wellbeing during the lockdown. The lack of access to equipment impacted on their ability to train effectively and the difficulty buying specific foods needed to fuel their recovery slowed down their progress. These stressors were further amplified by the lack of access to their usual coping resources particularly social support. The literature suggests that upon removal of athletes sporting platforms for example through retirement or injury, athletes lose access to vital coping resources such as social support which can threaten their wellbeing (Jewett et al., 2019). Emerging research suggests that the COVID-19 crisis and lockdowns have caused athletes to experience a similarly, enforced pause in their athletic career to that of forced retirement and chronic injury (Jewett et al., 2019; Gupta and McCarthy, 2021). Therefore, it has been suggested that career transition support and chronic injury psychological rehabilitation strategies be used to guide interventions with athletes who are struggling to cope with this non-normative transition.

Stambulova et al. (2020) suggests that COVID-19 can be interpreted as a “career transition barrier” that interferes with athletes’ ability to strive towards “career excellence.” The present study findings suggest that from a holistic developmental perspective (Wylleman, 2019), the social distancing measures impacted participants’ athletes, psychological, psychosocial, academic-vocational and financial development. Some athletes adapted their training routines to cope with this non-normative transition, whilst others experienced a crisis transition due to lack of resources and ineffective coping strategies. For example, some developed maladaptive behaviours such as disordered eating, sleep disruption, body image disturbance, depression and anxiety. These findings support the recommendation of Stambulova et al. (2020) that athletes need professional support in order to successfully negotiate this transition.

Samuel et al. (2020) conceptualised the COVID-19 pandemic as a “longitudinal, multifaceted, unpredicted, non-controlled change-event, with four distinct stages: (a) a pre-Coronavirus stage with unique career contextual conditions (i.e., stable engagement or a transitional period), (b) Coronavirus stage-A accompanied by instability and confusion, emotional response, and cognitive appraisal, (c) Coronavirus stage-B characterized by active coping or regression, and (d) Coronavirus stage-C; instability endures or decreases, depending on career trajectory.” (pg1). The present study findings provide empirical evidence to support these proposed stages, as the athletes negotiated this career change-event by implementing changes across a number of the proposed dimensions, and recommend strategies such as setting new goals to help boost motivation, adapting their athletic career aspirations, developing new social networks outside of sport and broadening their self-identity in order to navigate the COVID-19 transition.

### Strategies to Protect Wellbeing

Whilst the current study suggests that athletes did experience threats to their wellbeing in response to the social distancing measures that restricted their participation in sport, they also developed strategies to protect it during the crisis. These included adapting training routines and setting new goals, utilising social support and exercising outdoors to boost mood. The athletes adapted their training programmes in order to provide a structure to their lives during the lockdown, and shifted their motivational focus from enhancing performance to avoiding performance decrements. These finding support the research by Mascret (2020) who reported that during the lockdown athletes modified their achievement goals from self-approach goals (improving oneself) to self-avoidance goals (avoiding regression). Similarly, Gupta and McCarthy (2021) reported that the incongruence experienced by athletes due to the lack of structure during the lockdown resulted in psychological distress, negative emotions and loss of motivation.

Social support was instrumental in protecting the athlete’s wellbeing during the lockdown, and they identified how different structural support systems provided specific functions, such as relying upon family members for emotional support and their coaches for feedback on their athletic accomplishments. Social support has been described in the literature as a multidimensional construct that is comprised of three interdependent dimensions; structural (who provides the support e.g., family, friends, coach, teammates), functional (how is the support experienced e.g., emotional, esteem, tangible, network and informational forms) and perceptual (appraisals of the available amount and quality of the social support sources) (Bianco and Eklund, 2001; Holt and Hoar, 2006). Together with the present findings, this evidence suggests that athlete support personnel working with athletes during periods of isolation, need to assess and identify their social support preferences to ensure they are consistent with their needs and enhance its impact.

The findings of the current study support previous research which suggests that physical activity can help protect wellbeing during lockdown, and this effect is amplified if the activity takes place outside. Indeed, Thompson-Coon et al. (2011) suggested that participating in physical activity in outdoor natural environments has a greater impact on physical and mental wellbeing than physical activity indoors (Thompson-Coon et al., 2011).

Many of the athletes in the current study were motivated to not just train to enhance their athletic performance but to exercise to boost their mood, as per previous research (Rosenbaum et al., 2014; Rebar et al., 2015; Bailey et al., 2018). However, in light of the current global crisis there is a growing concern that social distancing measures that restrict exercise will contribute to a rise in obesity, depression, infections and cardiovascular disease (Chen et al., 2020). Furthermore, Mehrsafar et al. (2020) suggested that a certain level of anxiety regarding the pandemic is completely normal, but that high levels of stress can have a devastating impact on daily life and the inability to manage stress can result in short- or long-term depression (Frank et al., 2020). Recent research conducted during the COVID-19 pandemic has reported that higher levels of physical activity were associated with lower levels of anxiety, suggesting that physical activity can help protect against some of the stressors associated with the crisis (Antunes et al., 2020), suggesting that our athletes’ efforts to exercise improved overall wellbeing and were not in vein.

### Engaging in Reflective Practice Encourages Stress Related Growth

The athletes in this study engaged in a process of reflective practice during the lockdown period and this helped facilitate “stress related growth” (Park et al., 1996) in response to the challenges presented by the pandemic. It has been suggested that not all stressful experiences result in negative consequences and that the process of confronting them can help broaden perspectives, develop new coping strategies, enhance relationships and mobilise personal resources (Park and Fenster, 2004). Results from the current study suggest that athletes in lockdown were afforded a prolonged period of wakeful resting (Eccles and Kazmier, 2019), that allowed them to reflect on their athletic participation and make life changes that would protect their wellbeing during the confinement period and when they returned to sport. These strategies included developing social support networks and engaging in activities and hobbies outside of their sport. In particular, they identified the importance of maintaining a dual career and how this was protective for their wellbeing during lockdown and this period encouraged them to consider their plans for retirement.

The recovery literature associates rest with the reduction or cessation of physical activity and is fundamental for physical and psychological recovery following training and competition (Kellmann et al., 2018). Athletes become well rested if they engage in the resting process which includes sleeping and wakeful resting (Eccles and Kazmier, 2019). Athletes experience wakeful resting when they do not think about their sport, for example through participating in activities outside of it. The ability to engage in wakeful rest that facilitates a period of “psychological inactivity” can be protective for athlete wellbeing (Eccles and Kazmier, 2019). Indeed, the athletes in the current study reflected on how this enforced hiatus had allowed them to take a much needed rest from the physical and mental stress of competition. Although, for some this was particularly challenging during the first few weeks of lockdown due to the loss of structure and routine in their lives.

Some athletes used this time out of competitive sport to recover from past injuries and acknowledged their willingness to play hurt. Schneider et al. (2019) suggested that participation in competitive sport despite being injured is risky, but a common occurrence in athletes. The authors reported that the older the athlete the higher the inclination for self-exploitation and self-endangerment as reflected in their willingness to “play hurt.” Others reflected on how the pause in their athletic schedule had allowed them to engage in non-sport related hobbies and build relationships with family and friends. Pink et al. (2015) suggested that a break from the pressures of sport can provide perspective to the individual and encourage the development of a multidimensional identity which can enhance wellbeing (Pink et al., 2018).

It has been suggested that the development of an adaptive and resilient personality, that enables athletes to control anxiety is fundamental to achieving success in high performance sport (Kellmann and Günther, 2000). It may be that in order to cope with the social distancing measures that pose a threat to athlete wellbeing, that particular personality traits could play a protective role. Indeed, the current research suggests that athletes engaged in a reflective practice process during the crisis and this helped them evaluate their situation and employ appropriate coping strategies. Indeed, recent research conducted with aspiring Olympic and Paralympic Spanish athletes, reported that the lockdown period did not increase anxiety levels perhaps due to their adaptive and resilient personality traits (Clemente-Suárez et al., 2020).

### Practical Implications and Future Research

The present study highlights athlete’s experiences of the COVID-19 pandemic, in particular, how lockdown restrictions have impacted athlete wellbeing. Findings of this study offer a number of more immediate practical recommendations. Firstly, the findings can be integrated into current athlete wellbeing strategies provided by athlete support personnel. It is paramount that intra and interdisciplinary collaboration between athlete support personnel is encouraged, in order to coordinate efforts to help athletes cope with the current global crisis. For example, case conferencing between multidisciplinary team members and key stake holders to identify athletes with pre-existing mental health problems, injuries or poor social networks who could be at increased risk during periods of social isolation and mobilise appropriate professional support. Sport psychology support should focus on the maintenance of wellbeing and the development of self-care strategies rather than performance gains during this crisis. Athletes are encouraged to adapt their athletic routines to facilitate a positive adjustment to the constraints of social distancing and help protect against the development of maladaptive coping behaviours. Goal setting strategies that focus on a mastery approach that encourages personal development over a performance approach that is focused on outperforming others (Elliot and McGregor, 2001), should be encouraged in order to boost motivation.

It is recommended that efforts should focus on developing social support within teammates and coaches through incorporating team activities, that provide an opportunity to check in and promote social connectedness. These might include virtual team competitions such as quiz nights or guided discussions to help encourage mutual sharing of their experiences. With the increased accessibility of virtual communication in response to the pandemic, this is an opportune time to capitalise on these new technologies to help enhance athlete wellbeing. In particular, it is worth evaluating the effectiveness of tele-consulting and future research is encouraged to investigate how practitioners can deliver effective psychological support through tele-consulting, and to consider whether their support is best focused on therapeutic counselling or mental skills training during the pandemic. Beyond the COVID-19 pandemic studies could explore how such technologies can be utilised to promote social connectedness during times of normal physical distancing such as injury and breaks between seasons.

Lastly, and in line with previous career transition research (Wylleman, 2019), athletes are encouraged to pursue dual careers to facilitate the development of multiple identities that can help protect wellbeing during times away from sport. In the very least athletes should be encouraged to participate in hobbies and activities outside of the sporting arena.

### Strengths and Limitations

Qualitative methods of enquiry provide rich data and facilitate in depth exploration and examination of issues (Anderson, 2010). This qualitative study used an inductive approach which enabled the researchers to explore the perceived impact of social distancing measures during the COVID-19 pandemic on athlete wellbeing. However, the utilisation of an explicit wellbeing scale may have provided further validation to the findings (Ryff, 1989).

This study has a number of key methodological strengths. First, the heterogonous sample (Braun and Clarke, 2013) included athletes of different ages and career stages, as well as representing individual and team sports, and from different countries. However, a potential limitation of this heterogeneity is that there may be different cross-cultural conceptions of wellbeing. The majority of the athletes in the study were white so future research must seek to include athletes from other racial backgrounds. Findings reported in this article are unique to this sample and this current pandemic, thus generalization of findings should be avoided, though it would be pertinent to conduct future research that surveys a larger number of athletes. Utilisation of the open photo elicitation method is another strength of this research. Having been previously used in sporting research (e.g., Wakefield and Watt, 2012), the use of visual imagery in research is thought to evoke memories and facilitate greater immersion and more detailed storytelling by participants, providing rich experiential data (Green and Brock, 2002). However, the interview process and our interpretations of the athlete’s stories could have been influenced by the authors experience and knowledge as a sport psychologist, although the participants were invited to verify the findings in order to decrease the risk of misinterpretation (Karnieli-Miller et al., 2009).

## Conclusion

In summary, the COVID-19 social distancing measures have had a considerable impact on athlete wellbeing. The initial sudden loss of sport in the athlete’s lives posed a threat to their wellbeing, but over the duration of the lockdown period the athletes developed numerous strategies to protect it, such as adapting their routines, setting new goals, fostering social connectedness through technology and participating in new hobbies. Furthermore, their time away from sport encouraged them to reflect on their athletic career and to make life changes that would protect their wellbeing should they experience any future lockdowns.

## Data Availability Statement

The raw data supporting the conclusions of this article will be made available by the authors, without undue reservation.

## Ethics Statement

The studies involving human participants were reviewed and approved by Research Ethics Committee, School of Social Sciences, Humanities and Law, University of Teesside. The patients/participants provided their written informed consent to participate in this study.

## Author Contributions

LW conceived the study and conducted participant interviews. LW conducted data analysis and interpretations were reviewed by LB and created the initial manuscript draft. LB provided critical revisions of successive drafts. LW and LB wrote and edited the manuscript. Both authors contributed to the article and approved the submitted version.

## Conflict of Interest

The authors declare that the research was conducted in the absence of any commercial or financial relationships that could be construed as a potential conflict of interest.

## References

[B1] Anderson C. (2010). Presenting and evaluating qualitative research. *Am. J. Pharm. Educ.* 74 141–141. 10.5688/aj7408141 21179252 PMC2987281

[B2] Antunes R. Amaro N. Salvador R. Matos R. Morouço P. Rebelo-Gonçalves R. (2020). Higher physical activity levels may help buffer the negative psychological consequences of COVID-19 pandemic. *Front. Psychol.* 12:672811. 10.3389/fpsyg.2021.672811 33967927 PMC8100311

[B3] Bailey A. P. Hetrick S. E. Rosenbaum S. Purcell R. Parker A. G. (2018). Treating depression with physical activity in adolescents and young adults: A systematic review and meta-analysis of randomised controlled trials. *Psychol. Med.* 48 1068–1083. 10.1017/S0033291717002653 28994355

[B4] Bates E. A. McCann J. J. Kaye L. K. Taylor J. C. (2017). “Beyond words”: A researcher’s guide to using photo elicitation in psychology. *Qual. Res. Psychol.* 14 459–481. 10.1080/14780887.2017.1359352

[B5] Beable S. Fulcher M. Lee A. C. Hamilton B. (2017). SHARPSports mental health awareness research project: Prevalence and risk factors of depressive symptoms and life stress in elite athletes. *J. Sci. Med. Sport* 20 1047–1052. 10.1016/j.jsams.2017.04.018 28601589

[B6] Bianco T. Eklund R. C. (2001). Conceptual considerations for social support research in sport and exercise settings: The case of sport injury. *J. Sport Exerc. Psychol.* 23 85–107. 10.1123/jsep.23.2.85

[B7] Brand R. Wolff W. Hoyer J. (2013). Psychological symptoms and chronic mood in representative samples of elite student-athletes, deselected student-athletes and comparison students. *School Ment. Health* 5 166–174. 10.1007/s12310-012-9095-8

[B8] Braun V. Clarke V. (2006). Using thematic analysis in psychology. *Qual. Res. Psychol.* 3 77–101. 10.1191/1478088706qp063oa 32100154

[B9] Braun V. Clarke V. (2013). *Successful qualitative research: A practical guide for beginners.* London: SAGE.

[B10] Braun V. Clarke V. (2019). Reflecting on reflexive thematic analysis. *Qual. Res. Sport Exerc. Health* 11 589–597. 10.1080/2159676X.2019.1628806

[B11] Braun V. Clarke V. (2020). One size fits all? what counts as quality practice in (reflexive) thematic analysis? *Qual. Res. Psychol.* 2020 1–25. 10.1080/14780887.2020.1769238

[B12] Braun V. Clarke V. (2021). Conceptual and design thinking for thematic analysis. *Qual Psychol.* 2021:196. 10.1037/qup0000196

[B13] Braun V. Clarke V. Weate P. (2016). “Using thematic analysis in sport and exercise research,” in *Routledge Handbook of Qualitative Research in Sport and Exercise*, eds Smith B. Sparkes A. C. (Abingdon: Routledge), 191–205.

[B14] Brooks S. K. Webster R. K. Smith L. E. Woodland L. Wessely S. Greenberg N. (2020). The psychological impact of quarantine and how to reduce it: rapid review of the evidence. *Lancet* 395 912–920. 10.1016/S0140-6736(20)30460-8 32112714 PMC7158942

[B15] Brown J. C. Kerkhoffs G. Lambert M. I. Gouttebarge V. (2017). Forced retirement from professional rugby union is associated with symptoms of distress. *Int. J. Sports Med.* 38 582–587. 10.1055/s-0043-103959 28564743

[B16] Busanich R. McGannon K. R. Schinke R. J. (2014). Comparing elite male and female distance runner’s experiences of disordered eating through narrative analysis. *Psychol. Sport Exerc.* 15 705–712. 10.1016/j.psychsport.2013.10.002

[B17] Chen P. Mao L. Nassis G. P. Harmer P. Ainsworth B. E. Li F. (2020). Coronavirus disease (COVID-19): the need to maintain regular physical activity while taking precautions. *J. Sport Health Sci.* 9 103–104. 10.1016/j.jshs.2020.02.001 32099716 PMC7031771

[B18] Clemente-Suárez V. J. Fuentes-García J. P. de la Vega Marcos R. Martínez Patiño M. J. (2020). Modulators of the personal and professional threat perception of olympic athletes in the actual COVID-19 crisis. *Front. Psychol.* 11:1985. 10.3389/fpsyg.2020.01985 32849157 PMC7419607

[B19] Drew M. Vlahovich N. Hughes D. Appaneal R. Burke L. M. Lundy B. (2018). Prevalence of illness, poor mental health and sleep quality and low energy availability prior to the 2016 summer Olympic games. *Br. J. Sports Med.* 52 47–53. 10.1136/bjsports-2017-098208 29056598

[B20] Duncan G. E. Avery A. R. Seto E. Tsang S. (2020). Perceived change in physical activity levels and mental health during COVID-19: Findings among adult twin pairs. *PLoS One* 15:e0237695. 10.1371/journal.pone.0237695 32790745 PMC7425865

[B21] Eccles D. W. Kazmier A. W. (2019). The psychology of rest in athletes: An empirical study and initial model. *Psychol. Sport Exerc.* 44 90–98. 10.1016/j.psychsport.2019.05.007

[B22] Elliot A. J. McGregor H. A. (2001). A 2 X 2 achievement goal framework. *J. Pers. Soc. Psychol.* 80 501–519. 10.1037/0022-3514.80.3.501 11300582

[B23] Facer-Childs E. R. Hoffman D. Tran J. N. Drummond S. P. A. Rajaratnam S. M. W. (2021). Sleep and mental health in athletes during COVID-19 lockdown. *Sleep* 44:zsaa261. 10.1093/sleep/zsaa261 33535229 PMC7928674

[B24] Frank A. Fatke B. Frank W. Förstl H. Hölzle P. (2020). Depression, dependence and prices of the COVID-19-crisis. *Brain Behav. Immun.* 87 99–99. 10.1016/j.bbi.2020.04.068 32360604 PMC7189841

[B25] Giles S. Fletcher D. Arnold R. Ashfield A. Harrison J. (2020). Measuring well-being in sport performers: where are we now and how do we progress? *Sports Med. (Auckland)* 50 1255–1270. 10.1007/s40279-020-01274-z 32103451 PMC7305091

[B26] Gouttebarge V. Aoki H. Lambert M. Stewart W. Kerkhoffs G. (2017a). A history of concussions is associated with symptoms of common mental disorders in former male professional athletes across a range of sports. *Phys. Sportsmed.* 45 443–449. 10.1080/00913847.2017.1376572 28870119 PMC9336050

[B27] Gouttebarge V. Hopley P. Kerkhoffs G. Verhagen E. Viljoen W. Wylleman P. (2017b). Symptoms of common mental disorders in professional rugby: An international observational descriptive study. *Int. J. Sports Med.* 38 864–870. 10.1055/s-0043-114010 28895619

[B28] Green M. C. Brock T. C. (2002). “Narrative impact: Social and cognitive foundations,” in *In the mind’s eye: Transportation-imagery model of narrative persuasion*, eds Green M. C. Strange J. J. Brock T. C. (Mahwah, NJ: Lawrence Erlbaum Associates Publishers), 315–341.

[B29] Greenleaf C. Petrie T. A. Carter J. Reel J. J. (2009). Female collegiate athletes: prevalence of eating disorders and disordered eating behaviors. *J. Am. Coll. Health.* 57 489–496. 10.3200/JACH.57.5.489-496 19254889

[B30] Gulliver A. Griffiths K. M. Mackinnon A. Batterham P. J. Stanimirovic R. (2014). The mental health of Australian elite athletes. *J. Sci. Med. Sport* 18 255–261. 10.1016/j.jsams.2014.04.006 24882147

[B31] Gupta S. McCarthy P. J. (2021). Sporting resilience during COVID-19: what is the nature of this adversity and how are competitive elite athletes adapting? *Front. Psychol.* 12:611261. 10.3389/fpsyg.2021.611261 33746833 PMC7966721

[B32] Henriksen K. Schinke R. Moesch K. McCann S. Parham W. D. Larsen C. H. (2019). Consensus statement on improving the mental health of high performance athletes. *Int. J. Sport Exerc. Psychol.* 8 553–560. 10.1080/1612197X.2019.1570473

[B33] Hogan S. Warren L. (2012). ‘Dealing with complexity in research processes and findings: How do older women negotiate and challenge images of aging?’. *J. Women Aging.* 24 329–350. 23098046 10.1080/08952841.2012.708589

[B34] Holloway I. Galvin K. (2017). *Qualitative research in nursing and healthcare*, Fourth Edn. Chichester, West Sussex: Wiley Blackwell.

[B35] Holt N. L. Hoar S. D. (2006). “The multidimensional construct of social support,” in *Literature review in sport psychology*, eds Hanton S. Mellalieu S. D. (London: Nova Science Publishers).

[B36] Jewett R. Kerr G. Tamminen K. (2019). University sport retirement and athlete mental health: a narrative analysis. *Qual. Res. Sport Exerc. Health.* 11 416–433. 10.1080/2159676X.2018.1506497

[B37] Jia R. Ayling K. Chalder T. Massey A. Broadbent E. Coupland C. (2020). Mental health in the UK during the COVID-19 pandemic: cross-sectional analyses from a community cohort study. *BMJ Open* 10:e040620. 10.1136/bmjopen-2020-040620 32933965 PMC7493070

[B38] Karnieli-Miller O. Strier R. Pessach L. (2009). Power relations in qualitative research. *Qual. Health Res.* 19 279–289. 10.1177/1049732308329306 19150890

[B39] Kellmann M. Bertollo M. Bosquet L. Brink M. Coutts A. J. Duffield R. (2018). Recovery and performance in sport: consensus statement. *Int. J. Sports Physiol. Perform.* 13 240–245. 10.1123/ijspp.2017-0759 29345524

[B40] Kellmann M. Günther K. D. (2000). Changes in stress and recovery in elite rowers during preparation for the olympic games. *Med. Sci. Sports Exerc.* 32 676–683. 10.1097/00005768-200003000-00019 10731012

[B41] Kiliç Ö Aoki H. Goedhart E. Hägglund M. Kerkhoffs G. M. M. J. Kuijer P. P. F. M. (2018). Severe musculoskeletal time-loss injuries and symptoms of common mental disorders in professional soccer: A longitudinal analysis of 12-month follow-up data. *Knee Surg. Sports Traumatol. Arthrosc.* 26 946–954. 10.1007/s00167-017-4644-1 28698928 PMC5847204

[B42] Kirby K. Moran A. Guerin S. (2011). A qualitative analysis of the experiences of elite athletes who have admitted to doping for performance enhancement. *Int. J. Sport Policy* 3 205–224. 10.1080/19406940.2011.577081

[B43] Kunimoto N. (2004). ‘Intimate archives: Japanese-Canadian family photography, 1939-1949. *Art. Hist.* 27 129–155. 10.1111/j.0141-6790.2004.02701005.x

[B44] Lundqvist C. (2011). Well-being in competitive sports—The feel-good factor? A review of conceptual considerations of well-being. *Int. Rev. Sport Exerc. Psychol.* 4 109–127. 10.1080/1750984X.2011.584067

[B45] Mann R. H. Clift B. C. Boykoff J. Bekker S. (2020). Athletes as community; athletes in community: Covid-19, sporting mega-events and athlete health protection. *Br. J. Sports Med.* 54 1071–1072. 10.1136/bjsports-2020-102433 32303522 PMC7497562

[B46] Markser V. Z. (2011). Sport psychiatry and psychotherapy. Mental strains and disorders in professional sports. Challenge and answer to societal changes. *Eur. Arch. Psychiatry Clin. Neurosci.* 261 182–185. 10.1007/s00406-011-0239-x 21901268

[B47] Mascret N. (2020). Confinement during covid-19 outbreak modifies athletes’ self-based goals. *Psychol. Sport Exerc.* 51:101796. 10.1016/j.psychsport.2020.101796 32922209 PMC7475734

[B48] Maxwell J. A. (2012). *A realist approach for qualitative research.* London: SAGE.

[B49] Mayan M. J. (2009). *Essentials of Qualitative Inquiry.* Walnut Creek, CA: Left Coast Press.

[B50] Mehrsafar A. H. Gazerani P. Moghadam Zadeh A. Jaenes Sánchez J. C. (2020). Addressing potential impact of COVID-19 pandemic on physical and mental health of elite athletes. *Brain Behav. Immun.* 87 147–148. 10.1016/j.bbi.2020.05.011 32387513 PMC7201218

[B51] Morse J. M. Barrett M. Mayan M. Olson K. Spiers J. (2002). Verification strategies for establishing reliability and validity in qualitative research. *Int. J. Qual. Methods* 1 13–22. 10.1177/160940690200100202

[B52] Nixdorf I. Frank R. Hautzinger M. Beckmann J. (2013). Prevalence of depressive symptoms and correlating variables among german elite athletes. *J. Clin. Sport Psychol.* 7 313–326. 10.1123/jcsp.7.4.313

[B53] Park C. L. Cohen L. H. Murch R. (1996). Assessment and prediction of stress related growth. *J. Pers.* 64:71. 10.1111/j.1467-6494.1996.tb00815.x 8656319

[B54] Park C. L. Fenster J. R. (2004). Stress-related growth: predictors of occurrence and correlates with psychological adjustment. *J. Soc. Clin. Psychol.* 23 195–215. 10.1521/jscp.23.2.195.31019

[B55] Park S. Lavallee D. Tod D. (2013). Athletes’ career transition out of sport: a systematic review. *Int. Rev. Sport Exerc.* 6 22–53. 10.1080/1750984x.2012.687053

[B56] Patton M. Q. (2002). Two decades of developments in qualitative inquiry: A personal, experiential perspective. *Qual. Soc. Work.* 1 261–283. 10.1177/1473325002001003636

[B57] Pillay L. Janse van Rensburg D. C. C. Jansen van Rensburg A. Ramagole D. A. Holtzhausen L. Dijkstra H. P. (2020). Nowhere to hide: The significant impact of coronavirus disease 2019 (COVID-19) measures on elite and semi-elite South African athletes. *J. Sci. Med. Sport.* 23 670–679. 10.1016/j.jsams.2020.05.016 32448749 PMC7235602

[B58] Pink M. Saunders J. Stynes J. (2015). Reconciling the maintenance of on-field success with off-field player development: A case study of a club culture within the Australian football league. *Psychol. Sport Exerc.* 21 98–108. 10.1016/j.psychsport.2014.11.009

[B59] Pink M. A. Lonie B. E. Saunders J. E. (2018). The challenges of the semi-professional footballer: A case study of the management of dual career development at a Victorian Football League (VFL) club. *Psychol. Sport Exerc.* 35 160–170. 10.1016/j.psychsport.2017.12.005

[B60] Proctor S. L. Boan-Lenzo C. (2010). Prevalence of depressive symptoms in male intercollegiate student-athletes and nonathletes. *J. Clin. Sport Psychol.* 4 204–220. 10.1123/jcsp.4.3.204

[B61] Public Health England. (2020). *Lonliness during Coronavirus.* Available Online at: https://www.mentalhealth.org.uk/coronavirus/loneliness-during-coronavirus (accessed March 31, 2021).

[B62] Reardon C. L. Hainline B. Aron C. M. Baron D. Baum A. L. Bindra A. (2019). Mental health in elite athletes: international olympic committee consensus statement (2019). *Br. J. Sports Med.* 53 667–699. 10.1136/bjsports-2019-100715 31097450

[B63] Rebar A. L. Stanton R. Geard D. Short C. Duncan M. J. Vandelanotte C. (2015). A meta-meta-analysis of the effect of physical activity on depression and anxiety in non-clinical adult populations. *Health Psychol. Rev.* 9 366–378. 10.1080/17437199.2015.1022901 25739893

[B64] Ronkainen N. J. Ryba T. V. (2018). Understanding youth athletes’ life designing processes through dream day narratives. *J. Voc. Behav.* 108 42–56. 10.1016/j.jvb.2018.06.005

[B65] Rosenbaum S. Tiedemann A. Sherrington C. Curtis J. Ward P. B. (2014). Physical activity interventions for people with mental illness: A systematic review and meta-analysis. *J. Sci. Med. Sport.* 18:e150. 10.1016/j.jsams.2014.11.161 24813261

[B66] Ryff C. D. (1989). Happiness is everything, or is it? explorations on the meaning of psychological well-being. *J. Pers Soc. Psychol.* 57 1069–1081. 10.1037//0022-3514.57.6.1069

[B67] Samuel R. D. Tenenbaum G. Galily Y. (2020). The 2020 coronavirus pandemic as a change-event in sport performers’ careers: Conceptual and applied practice considerations. *Front. Psychol.* 11:567966. 10.3389/fpsyg.2020.567966 33071895 PMC7540073

[B68] Schinke R. Papaioannou A. Henriksen K. Si G. Zhang L. Haberl P. (2020). Sport psychology services to high performance athletes during COVID-19. *Int. J. Sport Exerc. Psychol.* 18 269–272. 10.1080/1612197X.2020.1754616

[B69] Schinke R. J. McGannon K. R. Smith B. (2013). Expanding the sport and physical activity research landscape through community scholarship: Introduction. *Qual. Res. Sport Exerc. Health* 5 287–290. 10.1080/2159676X.2013.847477

[B70] Schinke R. J. Stambulova N. B. Si G. Moore Z. (2018). International society of sport psychology position stand: Athletes’ mental health, performance, and development. *Int. J. Sport Exerc. Psychol.* 16 622–639. 10.1080/1612197X.2017.1295557

[B71] Schneider S. Sauer J. Berrsche G. Löbel C. Schmitt H. (2019). Playing hurt“– competitive sport despite being injured or in pain. *Deutsche Zeitschrift Für Sportmedizin* 2019 43–52. 10.5960/dzsm.2019.365

[B72] Schuring N. Kerkhoffs G. Gray J. Gouttebarge V. (2017). The mental wellbeing of current and retired professional cricketers: An observational prospective cohort study. *Phys. Sportsmed.* 45 463–469. 10.1080/00913847.2017.1386069 28952405

[B73] Şenışık S. Denerel N. Köyaðasıoðlu O. Tunç S. (2020). The effect of isolation on athletes’ mental health during the COVID-19 pandemic. *Phys. Sportsmed.* 49 187–193. 10.1080/00913847.2020.1807297 32762510

[B74] Sparkes A. C. (1998). Athletic identity: an Achilles’ Heel to the survival of self. *Qual. Health Res.* 8 644–664.

[B75] Stambulova N. B. Schinke R. J. Lavallee D. Wylleman P. (2020). The COVID-19 pandemic and Olympic/Paralympic athletes’ developmental challenges and possibilities in times of a global crisis-transition. *Int. J. Sport Exerc. Psychol.* 2020 1–10. 10.1080/1612197X.2020.1810865

[B76] Stanton R. To Q. G. Khalesi S. Williams S. L. Alley S. J. Thwaite T. L. (2020). Depression, anxiety and stress during COVID-19: Associations with changes in physical activity, sleep, tobacco and alcohol use in Australian adults. *Int. J. Environ. Res. Public Health.* 17:4065. 10.3390/ijerph17114065 32517294 PMC7312903

[B77] Swann C. Moran A. Piggott D. (2015). Defining elite athletes: Issues in the study of expert performance in sport psychology. *Psychol. Sport Exerc.* 16 3–14. 10.1016/j.psychsport.2014.07.004

[B78] Taylor S. (2019). *The psychology of pandemics: Preparing for the next global outbreak of infectious disease.* Newcastle upon Tyne: Cambridge Scholars Publishing.

[B79] Thompson-Coon J. Boddy K. Stein K. Whear R. Barton J. Depledge M. H. (2011). Does participating in physical activity in outdoor natural environments have a greater effect on physical and mental wellbeing than physical activity indoors? A systematic review. *Environ. Sci. Technol.* 45 1761–1772. 10.1021/es102947t 21291246

[B80] Tomalski J. Clevinger K. Albert E. Jackson R. Wartalowicz K. Petrie T. A. (2019). Mental health screening for athletes: Program development, implementation, and evaluation. *J. Sport Psychol. Action.* 10 121–135. 10.1080/21520704.2019.1604589

[B81] Toresdahl B. G. Asif I. M. (2020). Coronavirus disease 2019 (COVID-19): considerations for the competitive athlete. *Sports Health.* 12 221–224. 10.1177/1941738120918876 32250193 PMC7222670

[B82] Tracy S. J. (2010). Qualitative quality: Eight “Big-tent” criteria for excellent qualitative research. *Qual. Inq.* 16 837–851. 10.1177/1077800410383121

[B83] Wakefield C. Watt S. (2012). There will always be a part of you that longs to return: The usefulness of photo elicitation in exploring experiences of an ironman triathlon. *Qual. Res. Psychol. Bull.* 14:40.

[B84] Wolanin A. Hong E. Marks D. Panchoo K. Gross M. (2016). Prevalence of clinically elevated depressive symptoms in college athletes and differences by gender and sport. *Br. J. Sports Med.* 50 167–171. 10.1136/bjsports-2015-095756 26782764

[B85] World Health Organization. (2004). *Promoting mental health: Concepts, emerging evidence, practice.* Geneva: World Health Organization.

[B86] World Health Organization. (2021). *Coronavirus Disease 2019 (COVID-19): WHO Coronavirus (COVID-19) Dashboard.* Available Online at: https://covid19.who.int/ (accessed March 22, 2021).

[B87] Wylleman P. (2019). “A developmental and holistic perspective on transiting out of elite sport,” in *APA handbooks in psychology series. APA handbook of sport and exercise psychology Sport psychology*, Vol. 1 eds Anshel M. H. Petrie T. A. Steinfeldt J. A. (Washington, D.C: American Psychological Association), 201–2016.

[B88] Zhong B. Huang Y. Liu Q. (2021). Mental health toll from the coronavirus: Social media usage reveals wuhan residents’ depression and secondary trauma in the COVID-19 outbreak. *Comput. Hum. Behav.* 114 106524–106524. 10.1016/j.chb.2020.106524 32836728 PMC7428783

